# Anti-diabetic potential of chromium histidinate in diabetic retinopathy rats

**DOI:** 10.1186/s12906-015-0537-3

**Published:** 2015-02-05

**Authors:** Mustafa Ulas, Cemal Orhan, Mehmet Tuzcu, Ibrahim Hanifi Ozercan, Nurhan Sahin, Hasan Gencoglu, James R Komorowski, Kazim Sahin

**Affiliations:** Department of Physiology, Faculty of Medicine, Firat University, P.O. Box 23119, Elazig, Turkey; Department of Animal Nutrition and Nutritional Disorders, Faculty of Veterinary Medicine, Firat University, P.O. Box 23119, Elazig, Turkey; Department of Biology, Faculty of Science, Firat University, P.O. Box 23119, Elazig, Turkey; Department of Pathology, Faculty of Medicine, Firat University, P.O. Box 23119, Elazig, Turkey; Scientific and Regulatory Affairs, Nutrition 21 Inc., 3 Manhattanville Road, Purchase, NY 10577 USA

**Keywords:** Diabetes, Chromium, Retinopathy, Glucose transporter proteins, Oxidative stress

## Abstract

**Background:**

Chromium (Cr) is commonly used as a complementary medicine for diabetes mellitus. Several studies suggest that Cr intakes may improve glucose metabolism and decrease oxidative stress. Therefore, we aimed to assess the effects of chromium histidinate (CrHis) supplementation using a range of reliable biomarkers of oxidative damage and histopathological changes in rats with diabetic retinopathy.

**Methods:**

Diabetes was induced with streptozotocin [(STZ), 55 mg/kg] by intraperitoneal injection in male Long-Evans rats. Three weeks after STZ injection, rats were divided into four groups, namely, untreated normal controls, normal rats receiving CrHis (110 μg/kg/day); untreated diabetics and diabetics treated with CrHis (110 μg/kg/day) orally for 12 weeks.

**Results:**

In the untreated diabetic group, levels of serum glucose, glycosylated haemoglobin (HbA1c), total cholesterol (TC) and retina malondialdehyde (MDA) were significantly increased, while expressions of retina insulin, and glucose transporter 1 (GLUT 1) and glucose transporter 3 (GLUT3) and level of serum insulin were decreased. CrHis supplementation was found to reduce the levels of glucose, HbA1c, total cholesterol and MDA and to improve the GLUT1, GLUT3 and insulin expressions in STZ-induced diabetic rats. CrHis prevents the changes in the expressions of GLUT1, GLUT3 and insulin and the level of MDA in the retina tissue, confirming the protective effect of CrHis supplementation against the retinopathy caused by STZ. Histopathologic findings suggest that the CrHis-treated diabetic group had normal retinal tissue appearance compared with the untreated diabetic group.

**Conclusions:**

These results verify that CrHis has critical beneficial effects against retinal complications. Although detailed studies are required for the evaluation of the exact mechanism of the ameliorative effects of CrHis against diabetic complications, these preliminary experimental findings demonstrate that CrHis exhibits antidiabetic effects in a rat model of diabetic retinopathy by regulating the glucose metabolism and suppressing retinal tissue damage.

## Background

Diabetic retinopathy is the most common diabetic eye disease and a leading cause of blindness in working-age population. The pathophysiology of diabetic retinopathy is complex and multifactorial. The pathogenic process involves intricate interactions between oxidative stress and hyperglycemia [1,2]. Oxidative stress, increasing in diabetic retina during hyperglycemia, is linked to the augmented retinal basement membrane thickening, which is the hallmark of microangiopathy seen in diabetic retinal disease. In addition, the retina is the neurosensorial tissue of the eye and is extremely rich in polyunsaturated lipid membranes. This feature makes it especially sensitive to reactive oxygen species and lipid peroxidation. Diabetes mellitus increases oxidative stress and induces vascular leakage and increased retinal vascular permeability, perhaps causing macular edema which correlates with vision loss in diabetic retinopathy patients [3-5]. A strong association between hyperglycemia-induced retinal oxidative stress and the development of retinal histopathology has been supported by studies in diabetic animals treated with multiple trace elements. Certain trace elements supplementation can inhibit diabetes-induced retinal abnormalities associated with increased oxidative stress and can be directly and/or indirectly regulated by their interrelated homeostatic mechanisms to ensure normal retinal function [6].

In a high energy-demanding tissue such as retina, the regulation of glucose uptake and its utilization is important for the maintenance of normal retinal function. It is well known that glucose uptake is regulated by glucose transporter proteins (GLUTs) and all mammalian cells contain one or more members of this GLUT protein family. Among these transporters the GLUT3 and GLUT1 isoforms have been expressed in retinal cells [7,8]. Glucose uptake into retina cells occurs across the blood-retinal barrier via the GLUT1 and GLUT3 transporters. Glucose uptake into cells can vary among cell types, and such differences might be important in determining which cells are adversely affected by hyperglycemia [7].

The incidence of diabetes mellitus is associated with various events such as obesity, increasing age, physical inactivity etc. Furthermore, certain nutritional deficiencies have also been associated with a higher incidence of diabetes mellitus [8,9]. Chromium (Cr) deficiency has been associated with elevated blood glucose levels and diabetes mellitus [9]. Chromium is required for optimal insulin activity and normal carbohydrate and lipid metabolism [9,10]. Moreover, Cr improves glucose utilization through cellular signal transduction and glucose transporters. Results from the trials show that Cr supplementation in patients [10] and animals with type 1 and type 2 diabetes can improve both glucose and insulin metabolism [11]; in contrast to some studies rejecting the importance of Cr in treatment of type 2 diabetes [12]. For example, Kleefstra et al. [12] reported that no significant differences were found over time between the control and Cr treatment in the form of chromium yeast groups for fasting plasma glucose levels, blood pressure, body fat percentage, weight, lipid profile, and insulin resistance. However, as a novel Cr chelate, chromium histidinate (CrHis) is absorbed better than other forms of Cr compounds tested in human subjects [13]. Anderson et al. [13] reported that that humans absorbed an average 3.1 mcg of Cr from the CrHis complex, compared to 1.8 mcg from chromium picolinate (CrPic), 0.4 mcg from chromium chloride and 0.2 mcg from chromium polynicotinate. However, the exact mechanism by which supplemental CrHis exerts hypoglycemic action in diabetic diabetic retinopathy has not been fully elucidated. Therefore, this study was specifically planned to evaluate the effect of CrHis, a form of Cr developed to enhance stability and absorption of Cr supplementation, on metabolic risk factors and to determine the effect on the retinal tissue damage status of diabetic retinopathy rats.

## Methods

### Animals

Twenty eight Long Evans rats, useful animal model for studying ocular diabetic complications, aged 8 weeks with 250 ± 20 g of body weight were obtained from Ertugrul Kilic (Yeditepe University, Turkey). All the animals were kept and maintained under the standard guidelines and housed in the Veterinary Control Institute (Elazig, Turkey). The animals were kept and maintained at 22 ± 2°C, humidity of 55% ± 5% and 12/12-hour light/dark cycle. The rats were allowed free access to feed [standard pellet diet] and drinking water. The experiment was approved by the Animal Care and Use Committee, University of Firat with ethics number: 2011-10-131. All procedures involving rats were conducted in strict compliance with relevant laws, the Animal Welfare Act, Public Health Services Policy, and guidelines established by the Institutional Animal Care and Use Committee of the University. The rats were weighed every week and at the end of the study. Blood sample was collected from the tail vein of each rat for the measurement of biochemical efficacy and safety markers.

## Materials

The CrHis (Nutrition 21, Purchase, NY) was given in the water and administered at a concentration of 110 μg/kg bw/d) to get 9.16 μg elemental Cr (kg body/d), which is an equivalent dose of 614 μg Cr for a 70-kg adult human based on a previous work [9]. The Cr concentration of the water provided the control group was negligible (<1 μg/L). The water provided the Cr-supplemented group was initially prepared as a solution containing 3000 μg CrHis/L of water. The CrPic-supplemented water was diluted to achieve the target Cr intake per group on the basis of measured water intake [14]. STZ was obtained from Sigma (St. Louis, MO). To induce experimental diabetes, STZ was dissolved in citrate buffer (pH 4.5) and injected once intraperitoneally at a dose of 55 mg/kg to the remainder of the animals. A control group was given citrate buffer via intraperitoneal injection.

### Experimental design and induction of diabetes

Fourteen rats were treated with STZ (55 mg/kg body weight) through intraperitoneal injection. All rats were then fasted for 16 hour prior to treatment, but they had access to drinking water. The animals were divided into 4 groups: group I (Control) rats received citrate buffer intraperitoneally and isotonic saline, orally; group II (Control + CrHis) rats were administered chromium histidinate orally (110 μg/kg body weight) daily for a period of four weeks; group III (Diabetic) rats received single injection of STZ (55 mg/kg body weight) intraperitoneally and were also given isotonic saline, orally for the duration of the study; group IV (Diabetic + CrHis) diabetic rats were administered chromium orally as chromium histidinate (110 μg /kg body weight) daily for a period of 12 weeks after the induction of diabetes. Body weight and blood glucose concentrations were monitored weekly. Blood was collected from the tail vein of the rats. Blood glucose was determined by one touch glucometer (ACCU-Check Active, Roche Diagnostics, Mannheim, Germany) after the injection for 72 h. Before STZ injection, glucose concentrations of study rats and controls were measured and compared. After the injection of STZ, animals that exhibited fasting glucose levels greater than 140 mg/dL were considered as neonatal STZ diabetic resembling diabetes mellitus in humans.

### Laboratory analyses

At the end of the experiment, blood samples were centrifuged at 3000 × g for 10 min and sera were separated. Serum glucose concentrations were measured by using ACCU-Chek Active (Roche Diagnostics, Basel, Switzerland). Serum insulin levels were measured with the Rat Insulin Kit (Linco Research, St Charles, MO) by enzyme-linked immunosorbent assay (ELISA, Elx-800, BioTek Instruments, Winooski, VT). Serum concentrations of total cholesterol (TC) were measured by diagnostic kits (Sigma Diagnostics, St Louis, MO). Blood glycosylated haemoglobin (HbA1c) was also measured by routine kit (Alfabiotech, Milano, Italy) using the autoanalyzer. After rats were sacrificed, both eyes were enucleated and frozen at −80°C for the measurements of MDA, GLUT1, GLUT3 and insulin. The retina MDA content was measured by high performance liquid chromatography (HPLC, Shimadzu, Tokyo, Japan) using a Shimadzu UV–vis SPD-10 AVP detector and a CTO-10 AS VP column in a mobile phase consisting of 30 mM KH2PO4 and methanol (82.5 + 17.5, v/v; pH 3.6) at a flow rate of 1.2 ml/min. Column effluents were monitored at 250 nm and the volume was 20 μl. The retina homogenate (10%, w/v) was prepared in 10 mM phosphate buffer (pH 7.4), centrifuged at 13,000 × g for 10 min at 4°C, and the supernatant was collected and stored at −80°C for MDA analysis [15]. For Western blot analyses protein extraction was performed by homogenizing the retina in 1 ml ice-cold hypotonic buffer A, containing 10 mM 2-[4-(2-Hydroxyethyl)-1-piperazinyl]ethanesulfonic acid [HEPES] (pH 7.8), 10 mM KCl, 2 mM MgCl2, 1 mM DTT, 0.1 mM EDTA, and 0.1 mM phenylmethylsulfonyl-fluoride (PMSF). The homogenates were added with 80 μl of 10% Nonidet P-40 (NP-40) solution and then centrifuged at 14,000 × g for 2 min. The precipitates were washed once with 500 μl of buffer A plus 40 μl of 10% NP-40, centrifuged, re-suspended in 200 μl of buffer C [50 mM HEPES [pH 7.8], 50 mM KCl, 300 mM NaCl, 0.1 mM EDTA, 1 mM dihiothreitol [DTT], 0.1 mM PMSF, 20% glycerol], and recentrifugedat 14,800 × g for 5 min. The supernatants were collected for determinations of GLUT-1, GLUT-3 and insulin according to the method described by Sahin et al. [16]. Equal amounts of protein (50 μg) were electrophoresed and subsequently transferred onto a nitrocellulosemembrane (Schleicher and Schuell Inc., Keene, NH, USA). Antibodies against GLUT-1, GLUT-3 and insulin (Santa Cruz Biotechnology, Inc., Santa Cruz, CA, USA) were diluted (1:1000) in the same buffer containing 0.05% Tween-20. Protein loading was controlled sing a monoclonal mouse antibody against β-actin (A5316; Sigma). Bands were analyzed densitometrically using an image analysis system (Image J; National Institute of Health, Bethesda, USA).

### Histopathology

After the eye extirpation, tissue (retina) of each rat was examined grossly. The tissue was removed for histologic study, washed with normal saline, and immersion-fixed in 10% buffered formalin immediately upon removal. They were gradually dehydrated, embedded in paraffin, cut into 5-μm sections, and stained with hematoxylin and eosin for histologic examination according to standard procedures.

### Statistical analysis

Data were analyzed statistically using one-way ANOVA. In the analyses for serum glucose HbA1c, total cholesterol, and insulin concentrations, the repeated statement was added in the general linear model. The group differences were attained by the Fisher's multiple comparison test [Statistical Package for the Social Sciences (SPSS), Version 17.0, Chicago, IL]. A P value of less than 0.05 was considered significant.

## Results

### Effect of CrHis upon body weight and blood analysis in the rats diabetic-induced by streptozotocin

STZ administration affected the levels of typical blood parameters characteristic for diabetes, which are also accepted values in diabetes diagnostic (glucose, insulin and HbA1c). Blood glucose, HbA1c and total cholesterol levels were significantly increased in untreated diabetic rats compared to control groups while insulin levels were decreased (*P <* 0.5). When diabetic retinopathy rats were treated with CrHis, significant increases in blood glucose, HbA1c, insulin and total cholesterol levels were observed in diabetic retinopathy rats. CrHis treatment also resulted in a significant decrease in mean serum total cholesterol concentration of diabetic retinopathy animals. Body weight was significantly decreased (*P* < 0.001) in the untreated diabetic rats when compared to control group. CrHis treatment significantly increased body weight (*P* < 0.001) compared to the untreated diabetic group (Table 1).

**Table 1:** **Effect of CrHis supplementation on biochemical parameters in diabetic rats**

**Parameters**	**Control**	**CrHis**	**STZ**	**STZ + CrHis**
Body weight (g)	330 (3.6)^bc^	340 (3.9)^b^	225 (3.5)^c^	250 (3.5)^a^
Glucose (mg/dL)	110 (2.3)^a^	100 (2.0)^a^	480 (8.0)^b^	290 (2.7)^c^
Insulin (*μ*U/mL)	47.4 (0.20)^a^	50.3 (0.22)^a^	20.2 (0.25)^b^	25.0 (0.25)^c^
Total cholesterol (mg/dL)	90 (0.64)^a^	80 (0.34)^a^	240 (0.90)^b^	218 (0.80)^c^
Glycosylated hemoglobin (mg/g)	0.29 (0.02)^a^	0.20 (0.01)^a^	0.82 (0.05)^b^	0.45 (0.03)^c^

Data are expressed as mean (SD). Superscripts denote significant differences (P < .05) between groups.

### Effect of CrHis upon retinal GLUT1 and GLUT3 and insulin expression in the rats diabetic-induced by streptozotocin

**Figure 1:**
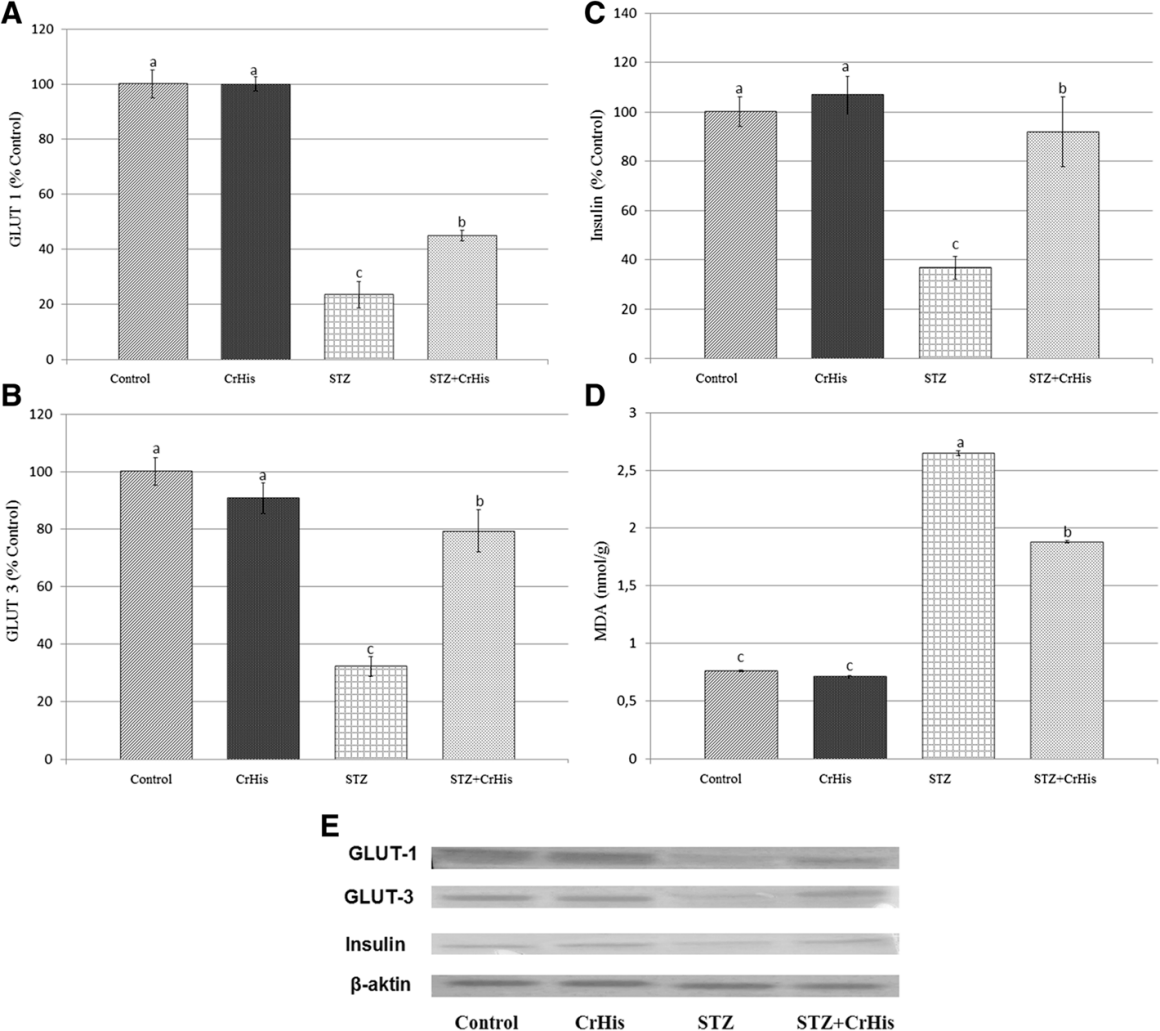
**Effect of chromium supplementation on the expression, GLUT1 (Panel A), GLUT3 (Panel B), insulin (Panel C), MDA (Panel D) values and density of proteins (Panel E) in the retina tissue regions of DR rats.** Data are means of quadruplets of assays and expressed as relative to control (%). Blots were repeated at least 3 times (n = 3) and a representative blot for each is shown. Actin was included to ensure equal protein loading. Values are LS means ± SE. Different letters within the retina parts indicate statistical differences among groups (p < 0.05).

Expressions of GLUT1, GLUT3 and insulin showed significant upward regulation (P < 0.05) in the retina of diabetic rats compared to control. Treatment using CrHis significantly (P < 0.05) reversed these changes to near control levels (Figure 1, Panel A-C).

### Effect of CrHis upon retinal MDA expression in the rats diabetic-induced by streptozotocin

The retina of untreated diabetic rats had considerably higher MDA expressions compared with controls (P < 0.001). A statistically significant reduction of MDA expression was found in retina of diabetic rats when the diabetic rats were treated with CrHis (Figure 1, Panel D).

### Histopthaology

**Figure 2:**
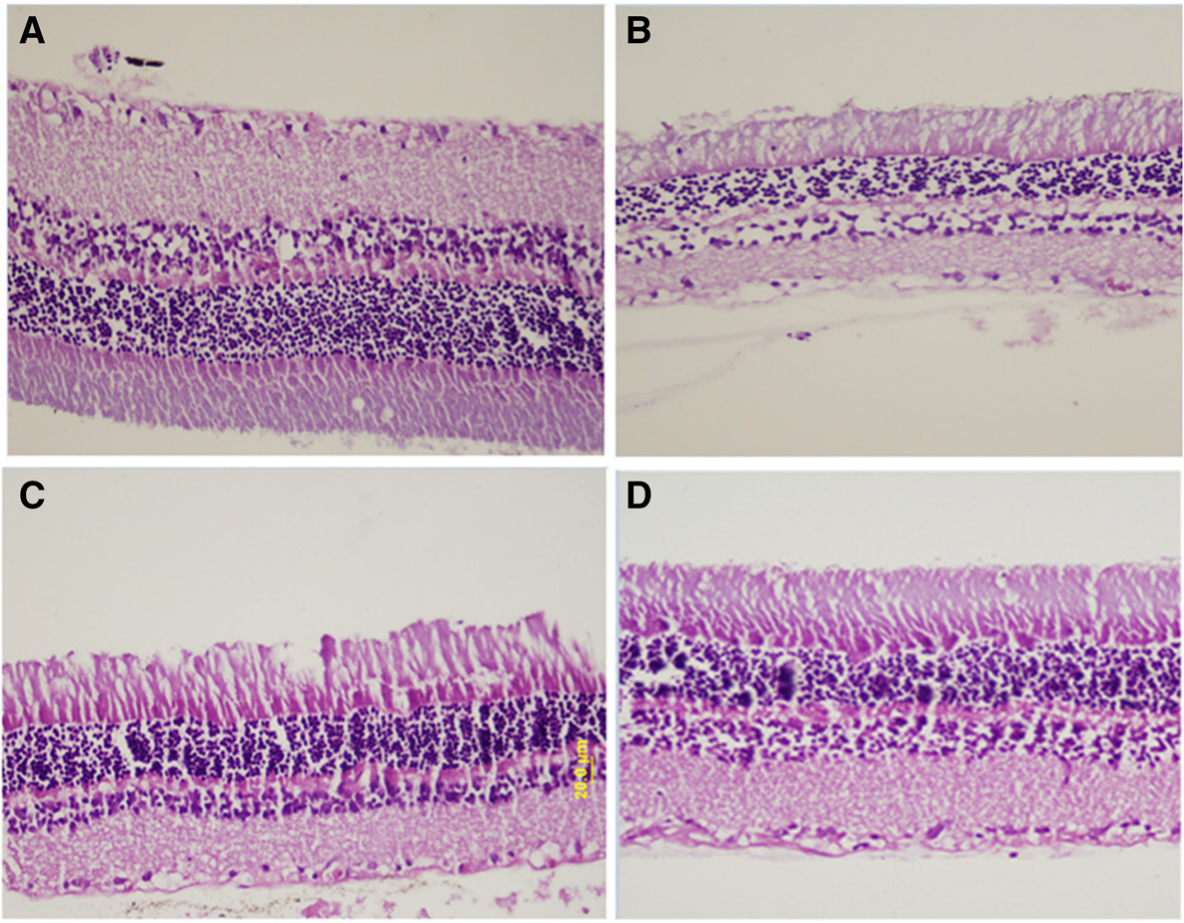
**Paraffin section photograph(s) of rat retina control and experimental group, control (A), CrHis (B), STZ + CrHis (C) and STZ (D) showing the histopathological changes.**

Retinas were highly organized in the normal (control) rats, with intact layers. The retinas were disorganized in the diabetic rats with impaired layers. But the retinas in CrHis group were improved compared to the diabetes group (Figure 2).

## Discussion

Diabetic retinopathy is the most common microvascular complication of diabetes. It remains a major cause of vision impairment or blindness in the world and high blood sugar level is considered the chief instigator in its development. The pathogenetic mechanisms of diabetic retinopathy are rather obscure. Many hyperglycemia-induced metabolic abnormalities are implicated in its pathogenesis, such as increased oxidative stress, and increased advanced glycation endproduct formation and other tissue proteins, which are observed during long-term hyperglycemia [6]. The retina has a high concentration of polyunsaturated fatty acids and the highest uptake of oxygen, which are vulnerable to lipid peroxidative damage. Free radical oxidation of the retinal photoreceptors adversely affects visual processing after the loss of lipoprotein membrane content [3,4]. Animal studies have demonstrated that oxidative stress, increasing in retina during hyperglycemia, is linked to the retinal capillary basement membrane thickening, which is one of the earliest abnormalities of the microangiopathy seen in diabetic retinopathy [4,5]. Hyperglycemia is generally recognized as a primary cause of diabetic complications and is associated with increased serum level of MDA [3,17]. In regards to diabetes mellitus, MDA levels are increased, indicating increasing lipid peroxidation. Diabetes induced oxidative stress can be responsible for the development of diabetic complications [17]. Trace elements and oxidative stress are associated with glycemic control and diabetic complications and trace elements supplementations such as Cr may help diabetic patients to slow the progression of this threatening complication of diabetes [9-11]. These findings were supported by a significant increase in MDA levels in retina tissue of diabetic retinopathy rats compared to other groups in our study. However, levels of MDA were significantly lower in the CrHis administration group and these research results were consistent with those obtained in other studies and in agreement that supplementation to diet of some essential trace metals such as Cr may play a role in the improvement of diabetes mellitus complications.

The risk of loss in visual acuity was correlated with the degree of retinal hard exudates, reducing serum lipid levels in patients with diabetic retinopathy [18]. The most common lipoprotein pattern in diabetes, also known as diabetic dyslipidemia, consists of an increase in triglyceride levels and decrease in high-density lipoprotein (HDL) cholesterol levels [19]. This is consistent with the study carried out by Yan et al. [20] to elucidate the relationship between diabetes and increase in serum cholesterol level. Faulkner et al. [21] clearly showed that the control of impaired glycaemic, which is defined by HbA1c, was proportionally related with degree of dyslipidemia. In addition, Cefalu [22] found that Cr supplementation improved glycemic control of type 2 diabetics by decreasing HbA1c% concentration. Jain et al. [23] also recorded that Cr is a key mineral for protection from heart diseases in streptozotocin-treated diabetic rats by reducing cholesterol levels in blood. In parallel with previous research, our study demonstrated that CrHis supplementation lowered total cholesterol level, and improved the HbA1c level in diabetic rats.

As one of the essential trace elements, trivalent cr plays a significant role in the regulation of glucose and insulin metabolism. Chromium is biologically active as part of an oligopeptide–chromodulin (low-molecular-weight chromium binding substance)–potentiating the effect of insulin by facilitating insulin binding to receptors at the cell surface. With Cr acting as a cofactor of insulin, Cr activity in the organism is parallel to insulin functions [24]. Cefalu et al. [25] stated that Chinese patients with type 2 diabetes receiving Cr experienced significant improvements in HbA1c, fasting serum glucose, 2-h glucose, and fasting and 2-h insulin. Similarly, Sahin et al. [9] reported that supplementary CrPic reduced blood glucose, total cholesterol levels and free fatty acid concentration and increased serum insulin levels and the composite insulin sensitivity index in rats. These results were also in agreement with Anderson et al. [26]. In addition Chen et al. [27] indicated that Cr supplementation has significant positive effects on glucose and insulin metabolism in patients with diabetes. This agreed with results of several authors such as Ravina et al. [28] who demonstrated that 10 days of treatment with CrPic (200 μg/Cr day) significantly increased insulin sensitivity in patients with type 1 or 2 diabetes and also enabled reductions in dosages of insulin and/or oral antidiabetic drugs in these patients. In the present study, the beneficial effect of Cr supplementation in diabetic retinopathy rats was observed in improvements in blood glucose and HbA1c levels. Complications of diabetic retinopathy were also shown to slow significantly while the same trend was observed for the carbohydrate metabolic profile as reflected in improved values of insulin expression and glucose transporter proteins.

Chromium has consistently indicated improvement in glucose transport in various tissues both in vitro and in animal models. Several mechanistic studies have demonstrated improvements in insulin receptor/postreceptor signaling, leading to enhanced activity of GLUTs. Interestingly, Cr can directly bind insulin, making the insulin more stable and possibly prolong its duration of action. Chromium treatment also helps the translocation of GLUTs and increases membrane fluidity by reducing the plasma membrane cholesterol [29]. The retina tissue has a high concentration of polyunsaturated fatty acids and high metabolic rates that require a constant and abundant supply of glucose from the circulation to meet their metabolic demands. Therefore, glucose uptake by this tissue plays an important role in determining glycemic control. In the retina, glucose is transported by GLUT1 [30] and GLUT3 [31] across the blood–retinal barrier. GLUT1 levels in the retina increase in relation to glucose transport and metabolism and tissue growth, whereas GLUT3 levels in neuroretina increase in relation to functional neuronal activity [32]. In the retina, endothelial cells express GLUT-1 [33], whereas neurons express GLUT3 [32]. Some studies have demonstrated that hyperglycemia plays a critical role in the pathogenesis of retinal disease. Klip et al. [34] indicated that whole-body glucose uptake is decreased in streptozocin-induced diabetic animals and in type 2 diabetic humans. In the diabetic retinopathy model, the observed lower expressions of GLUT1 and GLUT3 in the retina indicate the cellular biological properties similar to those of a combination of neurons and endothelial cells. Moreover, CrHis treatment effectively reversed this decrease in the retina. Increased GLUT1 and GLUT3 expressions in retina tissues upon hyperglycemia may be a response to compensate glucose need (Figure 1). Chromium serves as a cofactor for insulin action and enhances insulin signaling in animal models [35]. As examples, Cr supplementation has alleviated insulin resistance, enhanced intracellular insulin signaling in high fat diet/STZ rats, depressed lipolysis in adipocytes and inhibited gluconeogenesis in rats. Another study also reported an inverse association of Cr intake with fasting insulin level and several studies have reported benefits of chromium supplementation on insulin sensitivity in adults with or without type 2 diabetes mellitus [9]. Chromium supplementation can alleviate blurred vision and improve poor insulin binding and impaired glucose intolerance in human and animals [36]. These observations suggest that CrPic supplementation improves insulin sensitivity and improves retinal function by recovering retinal chromium concentration. Chromium can modulate the activity of insulin by increasing the insulin-sensitive cell receptors or binding activity and enhancing intracellular insulin signaling activity [37]. Insulin stimulates cellular glucose uptake in various tissues by inducing the translocation of GLUT from an intracellular pool to the plasma membrane [38]. The hypoglycemic effect of trivalent chromium was reported under insulin-deficient conditions [39] and exhibited significant antidiabetic potential in STZ-induced diabetes in rats. Our findings demonstrate that diabetic retinopathy syndrome in rats is associated with a reduction in retina tissue insulin levels, which reflects impairment in glucose uptake. This is further supported by other blot test examinations of retina tissues. Treatment of diabetic retinopathy rats with CrHis succeeded to normalize the lower retina insulin level. This may explain the reported effectiveness of CrHis treatment on the retina tissue levels of insulin.

## Conclusions

In conclusion, the results of the present study demonstrate the benefits of supplementation with CrHis in rats with diabetic retinopathy. CrHis supplementation offer improvements to markers of disease risk, including hyperglycemia insulin sensitivity, and increased oxidative stress. These findings are encouraging and warrant further clinical trials of this promising CrHis supplementation.

## Abbreviations

CrHis: Chromium histidinate
CrPic: Chromium picolinate
GLUT: Glucose transporter proteins
STZ: Streptozotocin
MDA: Malondialdehyde
HbA1c: Glycosylated haemoglobin
HDL: High-density lipoprotein
LDL: Low-density lipoprotein
ELISA: Enzyme-linked immunosorbent assay
PMSF: Phenylmethylsulfonyl-fluoride
HPLC: High performance liquid chromatography
HEPES: 2-[4-[2-Hydroxyethyl]-1-piperazinyl]ethanesulfonic acid
DTT: Dihiothreitol
ANOVA: Analysis of variance
SPSS: Statistical Package for the Social Sciences
